# Current News

**Published:** 1899-05

**Authors:** 


### Current News.

THE REVISED AND COMPLETE PROGRAMME OF THE AMERICAN MEDICAL ASSOCIATION, SECTION ON STOMATOLOGY.

Chairman's Address, Dr. G. V. I. Brown, Milwaukee.

"The Human Face and Jaws as a Danger Signal of Systemic Defect or Disorder," Dr. J. G. Kiernan, Chicago.

"Cocaine and Eucaine; Their Relative Toxicity," Dr. A. H. Peck, Chicago.

"Epithelial Structures in the Peridental Membrane," Dr. Frederick Noyes, Chicago.

"Infectious Ulcerative Stomatitis," Dr. John Marshall, Chicago.

"Oral Surgical Operation" (with illustrations showing remarkable results), Dr. G. V. I. Brown, Milwaukee.

"Some Points on the Etiology, Pathology, and Treatment of Persistent Pyorrhœa Alveolaris," Dr. G. T. Carpenter, Chicago.

"Interstitial Gingivitis" (so-called Pyorrhœa Alveolaris), giving the result of original work, with large photographic illustrations showing the progress of the disease from the beginning to the exfoliation of the teeth, Dr. Eugene S. Talbot, Chicago.

"Syphilitic Infection from Dental Instruments, with Cases," Dr. W. L. Baum, Chicago.

"Professional Education and Ethics," Dr. A. E. Baldwin, Chicago.

"Neuralgias due to Progressive Periosteal Necrosis," Dr. M. H. Fletcher, Cincinnati.

"The Treatment and Positive Cure of Pyorrhœa Alveolaris in Connection with Restoration of Normal Articulation," Dr. W. G. A. Bonwill, Philadelphia, Pa.

Dr. Bonwill will hold clinics independently of the meeting of the Section on Stomatology to those who wish to meet him.

G. V. I. Brown, *Chairman,*
Eugene S. Talbot, *Secretary.*

---

### THE NATIONAL DENTAL ASSOCIATION—COMMITTEE ON HISTORY.

Dear Doctor,—At the first meeting of the Association a committee was appointed to report a *measure looking to the preparation* of a full history of the dental profession. This committee will make a report at the meeting next August, and the character of the report will depend somewhat on the interest taken in this important subject by the members of this Association and the profession generally.

All must admit the necessity for a full, carefully prepared, and *authoritative* history of dentistry. The time (as the century closes) is most propitious, and the longer it is delayed the more difficult it will be to secure a reliable result.

The committee will be greatly helped in making its report by any interest you may take in this matter, and would be glad to have your replies to the following queries, together with any advice, suggestions, or objections you may be pleased to give.

1. Will you please name any books, pamphlets, manuscript reports, in fact, any matters of interest you may possess, which, at the proper time, might be available for the history?

2. Will you give the names and addresses of any dentists in your vicinity who have written on the subject or are interested in dental history?

3. Should the proposed work, in your opinion, be confined to a history of the profession in America, or should it be of dentistry from the earliest times all over the world?

4. As it is necessary for us to report on the probable financial success of the idea, would you be willing to subscribe, at the proper time, for a satisfactory history of the dental profession?

CHARLES McMANUS,
*Chairman.*

80 PRATT STREET, HARTFORD, CONN.

---

### DENTAL COMMISSIONERS OF CONNECTICUT—LEGAL NOTICE.

THE Dental Commissioners of Connecticut will meet in the Supreme Court Rooms at the Capitol in Hartford, Monday, May 15, 1899, at ten o'clock, to examine candidates for license and attend to all matters proper to come before them. Persons desiring to practise in this State must apply to the Recorder for proper blanks, which they will fill out and return to him before the day of examination.

GEORGE L. PARMELE,
*Dental Commissioner and Recorder.*

HARTFORD, April 10, 1899.

---

### DENTAL ASSOCIATION OF NEW SOUTH WALES.

THE Fifth Annual Meeting of the Dental Association of New South Wales was held at the Australia Hotel on Tuesday evening last, and was well attended. Dr. A. Burne, President, occupied the chair.

The President's Report for the past year dealt (among other matters) with the present position of the Dental Bill and the untiring work of the Council in its struggle to get at least eight clauses of the bill passed in a House talking nothing but federation.

The members expressed their confidence in the Council and appreciation of their endeavors, and felt confidence in the final result of the Bill being passed.

The balance sheet being read showed a balance in hand of £97 17*s.* 3*d.*, which, after deducting all the heavy expenses incurred, proved very satisfactory.

The following officers were elected for the year 1899–1900: President, Dr. A. Burne; Vice-Presidents, Messrs. H. Paterson and S. Chaim; Hon. Treasurer, Dr. O. Davis; Committee, Messrs. C. C. Marshall, H. S. Newton, E. A. Gabriel, and J. S. Darton; Auditors, Messrs. B. Corbett and C. Chandler; Hon. Secretary, Mr. H. Taylor.

The President (Dr. Burne), in returning thanks for his re-election, pointed out the present position of the Dental Bill and the prospects of its finally becoming law in the near future. He also expressed thanks to the Council and secretary for their support during a very trying term of office.

A vote of thanks to the chairman closed the meeting.

H. TAYLOR,
*Secretary.*

---

### SOUTH DAKOTA STATE DENTAL SOCIETY.

THE Seventeenth Annual Meeting of the South Dakota State Dental Society will be held in Yankton, on Wednesday, Thursday, and Friday, June 7, 8, and 9, 1899.

C. S. BLUNT,
*Secretary.*

